# Performance and skill retention of five supraglottic airway devices for the pediatric difficult airway in a manikin

**DOI:** 10.1007/s00431-018-3134-x

**Published:** 2018-04-05

**Authors:** Johannes Kulnig, Lisa Füreder, Nicole Harrison, Michael Frass, Oliver Robak

**Affiliations:** 1 Department of Pediatrics, University Hospital Tulln, Karl-Landsteiner University of Health Sciences, Tulln, Austria; 2 Department of Anaesthesia, General Intensive Care and Pain Medicine, Medical University of Vienna, Vienna, Austria; 3 Department of Medicine I, Medical University of Vienna, Waehringer Guertel 18-20, 1090 Vienna, Austria

**Keywords:** Supraglottic airway, Airway management, Intubation, Difficult airway, Pediatrician

## Abstract

Supraglottic airway devices (SADs) have been introduced to assist medical professionals in emergency situations with limited experience in securing airways via conventional endotracheal intubation (ETI). Literature on the use of SADs for securing an airway during pediatric critical settings is scarce, and there is a lack of studies comparing different SADs to each other and to conventional ETI. We conducted a study comparing five different SADs to ETI with regard to success rate, time to first ventilation, and personal rating in a pediatric manikin under simulated physiologic and pathologic airway conditions in 41 pediatricians of varying clinical experience and training. Only the AirQ, AuraG, and laryngeal tube (LT) were inserted within 30 s correctly by all participants under physiologic conditions. In tongue edema (TE), AirQ and LT had the highest success rate. In limited mobility of the cervical spine (CS), AirQ, AuraG, and LT again all were inserted within 30 s. In a multivariate analysis, factors influencing the success were experience with the respective device and level of medical education. Under TE conditions, there were significantly longer insertion times for the ETI, laryngeal mask airway (LMA), and EzT. Under CS conditions, there were significantly longer insertion times for the ETI, LMA, LT, and EzT. A multivariate analysis showed experience with the respective device to be the only factor of influence on time to first ventilation.

*Conclusion*: LT, AuraG, and AirQ were superior in providing fast and effective ventilation during simulated difficult airway situations in pediatricians.

**Table:** 

**What is Known:** • *Supraglottic airway devices have been introduced for medical professionals who lack experience for managing difficult airway situations*. • *A variety of these devices have been developed so far, but not compared to each other yet*.
**What is New:** • *We compared five different supraglottic airway devices with regard to success rate, time to first ventilation, and personal rating in a pediatric manikin under simulated physiologic and pathologic airway conditions*. • *Laryngeal tube, AuraG, and AirQ were superior in providing fast and effective ventilation during simulated difficult airway situations in pediatricians with varying clinical experience*.

## Introduction

In the setting of a pediatric emergency, pediatricians must be able to provide basic airway management. The current gold standard for securing an airway is endotracheal intubation (ETI) [7, 15]. The skills required to perform ETI successfully are usually acquired in training, but many pediatricians do not use them frequently. Because only a small percentage (5–10%) of out-of-hospital EMS calls is for pediatric patients, also many paramedics may have limited experience in working with children. Patient outcomes after pediatric cardiopulmonary arrest are dismal and have not improved over the last three decades [25]. Nonetheless, when an emergency occurs, the best chance for intact survival of the child is determined by adequate airway management.

Latest European Resuscitation Council (ERC) guidelines for resuscitation recommend that only skilled and experienced practitioners should perform ETI [14]. As a consequence, supraglottic airway devices (SADs) have been introduced for use by many medical professionals who lack experience in difficult airway situations as an effective way for managing difficult airway situations [23, 24]. These may be defined as clinical situations, in which a trained anesthesiologist experiences difficulties with bag-mask ventilation, difficulties with ETI, or both [3]. In the past decades, a variety of these devices have evolved, each possessing advantages and disadvantages in design and in clinical application [1, 22]. Presently, SADs are seen in almost every emergency cart worldwide.

Pediatric CPR on pediatric wards is often performed by pediatricians, who lack critical experience and adequate training for success during high-stress medical emergencies requiring the use of these devices. Given these facts, the devices used for managing a difficult airway situation should be simple, intuitive to use, and reliable. Unfortunately, the equipment used in clinical emergency carts often is determined by others than those who are required to attend to medical emergencies. Often, choices are made that are not based on clinical evidence. Preference of a specific airway device in a clinical emergency also depends, in part, on individual experience and training. We have chosen the five different SADs because of the following reasons: While use of the classic laryngeal mask airway (LMA, LMA™ Company North America, San Diego, CA, USA) is limited in emergencies because of leakage at relatively low pressures, it is one of the most popular and widespread devices in anesthesia. The Laryngeal tube (LT, VBM GmbH, Sulz, Germany) is widespread in the field of emergency intubation. The EasyTube® (EzT, Well Lead Medical Co., Guangzhou, China) is a further development of the Combitube which is a well-recognized emergency airway especially in the USA and Canada. AirQ® (Clearwater, FL, USA) is a masked laryngeal airway which might have some advantages over the LMA such as a better seal. Finally, the AuraGain® (AuraG, Ambu A/S, Ballerup, Denmark) resembles an LMA with the additional advantage of an integrated gastric access. We believe that these devices are equally effective and may have a similar degree of difficulty or complexity. We did not investigate bag mask ventilation (BMV) because of danger of aspiration. Furthermore, ventilation via BMV is suggested to be more difficult than other techniques. Previous studies have demonstrated that some SADs might provide superior benefits when used under difficult airway conditions in adult manikins, including swollen tongue, trismus, or limited movement of the cervical spine [18, 20]. A recent meta-analysis on SADs in pediatric anesthesia also showed possible benefits [13]. However, the latter do not comprise studies in pediatric difficult airway situations. Literature on the use of SADs for securing an airway during pediatric emergencies is scarce, and there is a lack of studies comparing different SADs to each other and to conventional ETI as the gold standard.

We therefore conducted a study comparing five different SADs with regard to success rate, time to first ventilation, and personal rating in a pediatric manikin under simulated physiologic and pathologic airway conditions with pediatricians of varying clinical experience and training. The investigated SADs have not been compared to each other yet as has been for adult airways. The aim of this study was to provide evidence for a recommendation regarding the use of SADs in pediatric difficult airway situations utilizing simulation of abnormal airway conditions.

## Materials and methods

Forty-one pediatricians of varying clinical experience and training participated in the present study. While it may appear unrealistic at first glance that non-trained pediatricians might be first-responders, it seemed important to us to investigate these SADs by non-trained doctors to avoid any prejudice if they would have used one of these SADs already in the past. This group of medical specialists was chosen due to the fact that usually pediatricians deal with pediatric emergences first until a hospital emergency team arrives. The study was approved by the local Ethical Committee (No. 1805/2015, date of approval 10/2015) and every participant agreed to his/her involvement in the study via declaration of informed consent prior to the study. Demographic data comprised age of service, sex, and amount of previous experience with SADs.

The present study utilized an airway simulation manikin (Laerdal SimJunior® 3G, Laerdal, Stavanger, Norway) to assess feasibility of placement and time to final placement of five supraglottic airway devices (LMA; AirQ, AuraG; LT, and EasyTube®) as well as conventional ETI utilizing an endotracheal tube (ET, Teleflex Medical GmbH, Belp, Switzerland). Two anesthesiologists with more than 10 years of experience in pediatric anesthesiology, who were not participants in the study, assessed correct placement as well as insertion time using a stopwatch and success of insertion and ventilation by inflation of the manikin’s lung.

All SADs were explained theoretically prior to the study. Explanation of each device lasted for 3 min. This step-by-step introduction comprised the distinct designs of the SADs, the method of insertion, inflation of cuffs and balloons, and method of ventilation. All participants were given the opportunity to get their hands on the devices and to take a closer look at each SAD. As it was considered that the use of the SADs were self-explanatory, the participants did not receive any additional practical training with the provided airway devices. Participants inserted every SAD under simulated standard physiologic airway conditions (STD) as well as under simulated pathological airway conditions such as tongue edema (TE), and limited mobility of the cervical spine (CS). Simulation of CS in a manikin model allowed us to mimic a patient with meningitis, traumatic neck injury, or seizure, all common complications, making it highly relevant in the management of a difficult airway. These conditions were chosen since they pose common life-threatening situations requiring immediate action and can identically be reproduced by the manikin [12]. The orders of the devices, as well as the order of the conditions, were randomized using a computerized random number generator (https://www.randomizer.org). To exclude any significant learning effect phenomenon, only a single insertion attempt was permitted, albeit some SADs were familiar to the participants [19].

The present study measured time from grasping the device to its final placement and the first ventilation. Cuffs were inflated, and the manikin was ventilated in order to confirm successful placement of the airway device. Attempts lasting longer than 30 s [6, 14], insufficient ventilation (judged by the manikin’s lung inflation), and termination of the placement of the SAD by the test person were classified as unsuccessful. Immediately after the study, the participants filled out a rating form (5 = worst performance, 1 = best performance) for every SAD under every given condition. The sizes of the SADs were chosen prior to the study according to their best fit to the manikin’s anatomy, which represents a 6-year-old child. Primary endpoint of the study was insertion success rates of the distinct devices; secondary endpoints comprised time to first ventilation and rating by the participants. After 6 months, a second run was performed to determine the training effect.

### Statistical analysis

Descriptive data is given as mean ± standard deviation. For statistical analysis of success rates of the distinct SADs under various simulated airway conditions, the McNemar’s test was used. For calculation of the grading of the SADs by the participants, we utilized the Wilcoxon signed-rank test. For analysis of the duration of the intubation attempt, the Student’s paired *t* test was utilized. All calculations were done with GraphPad Prism 5.0 software. *P* values < 0.05 were considered statistically significant.

To calculate the sample size, we determined the effect size (as difference between two groups, based on preceding data or data from the literature, respectively) and the corresponding standard deviation (for continuous data); in the absence of preceding data, effect size was set to 0.8 for sample size calculation. The power of the experiments to detect the postulated effect was set to 0.8 (1 − *β*, where *β* is the probability of committing a type II error, concluding that no difference between treatment groups exists, when there is a difference), at a significance level of 0.05. Using these values, the necessary sample size for the experiment was then calculated using G*Power 3.1 or PS Power and Sample Size Calculations 3.0 (two-tailed *t* test; Cohen’s *d* 0.8, alpha error 0.05, power 0.8). Estimates of required sample size were corrected for repeated measurements; if necessary, at least 28 participants were required.

## Results

All 41 participants (16 female) completed the study. All rating forms were filled out correctly. Mean clinical experience in pediatrics was 6.3 years (± 5.3). Demographic data and participants’ experiences with the provided SADs are provided in Table 1.

**Table 1:** Demographics and experience of participants

Variable	Number	Percent (or SD)
Participants (female)	41.00	39.02%
Age	32.78	6.09
Work experience (years)	6.29	5.27
Medical education		
Intern	13	31.71%
Resident	18	43.90%
Senior physician	10	24.39%
Experience with		
ETI	33	80.49%
LMA	19	46.34%
AirQ	0	0.00%
AuraG	0	0.00%
LT	8	19.51%
EzT	7	17.07%

### Success rate

Distinct success rates of the LMA, AirQ, AuraG, LT, and EzT under physiologic conditions are given in Table 2. Of note, only the AirQ, AuraG, and LT were inserted within 30 s correctly by all participants under physiologic conditions. In TE, AirQ and LT had the highest success rate. In CS, AirQ, AuraG, and LT again all were inserted within 30 s. In a multivariate analysis, factors influencing the success were experience with the respective device and level of medical education, but not work experience, age, or gender.

**Table 2:** Success rates and skill retention of conventional endotracheal tube (ET), Laryngeal Mask Airway (LMA), AirQ, AuraGain (AuraG), Larynx Tube (LT), and EasyTube (EzT) under physiologic (phys) conditions, tongue edema (TE), or immobilization of the cervical spine (CS)

1st run	ET		LMA		AirQ	
Success rate						
Physiologic conditions	36	87.80%	39	95.12%	41	100.00%
Tongue edema	9	21.95%	29	70.73%	41	100.00%
Cervical immobilization	22	53.66%	34	82.93%	41	100.00%
2nd run	ET		LMA		AirQ	
Success rate						
Physiologic conditions	37	90.24%	41	100.00%	41	100.00%
Tongue edema	12	29.27%	31	75.61%	41	100.00%
Cervical immobilization	22	53.66%	35	85.37%	41	100.00%
1st run	AuraG		LT		EzT	
Success rate						
Physiologic conditions	41	100.00%	41	100.00%	33	80.49%
Tongue edema	37	90.24%	41	100.00%	21	51.22%
Cervical immobilization	41	100.00%	41	100.00%	4	9.76%
2nd run	AuraG		LT		EzT	
Success rate						
Physiologic conditions	41	100.00%	41	100.00%	38	92.68%
Tongue edema	40	97.56%	41	100.00%	26	63.41%
Cervical immobilization	41	100.00%	41	100.00%	18	43.90%

### Time to first ventilation

**Fig. 1:**
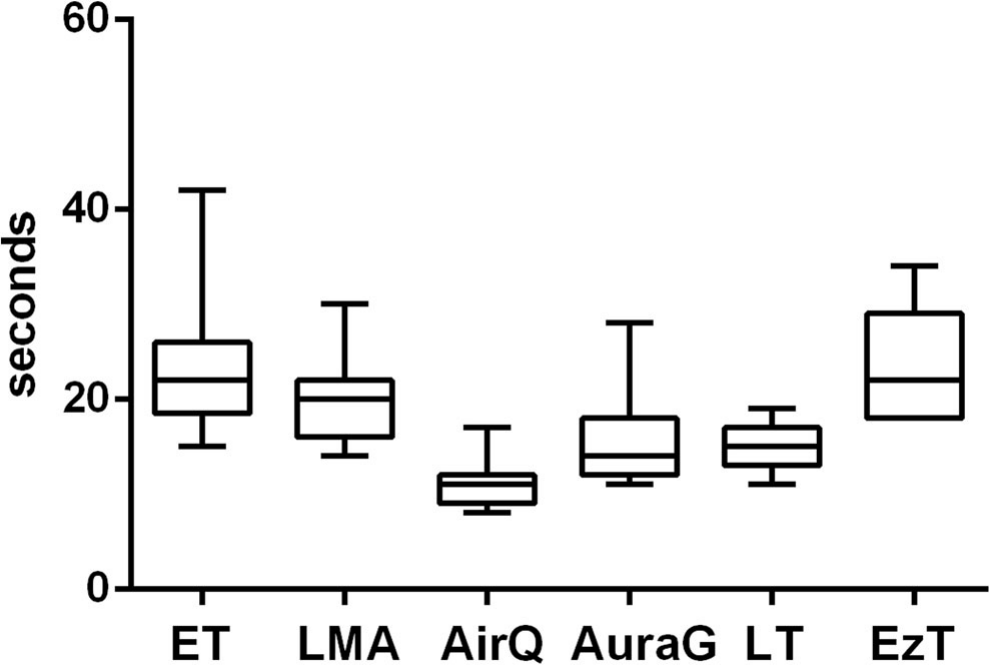
Time to first ventilation under physiologic conditions with ET, LMA, AirQ, AuraG, LT, and EzT

**Fig. 2:**
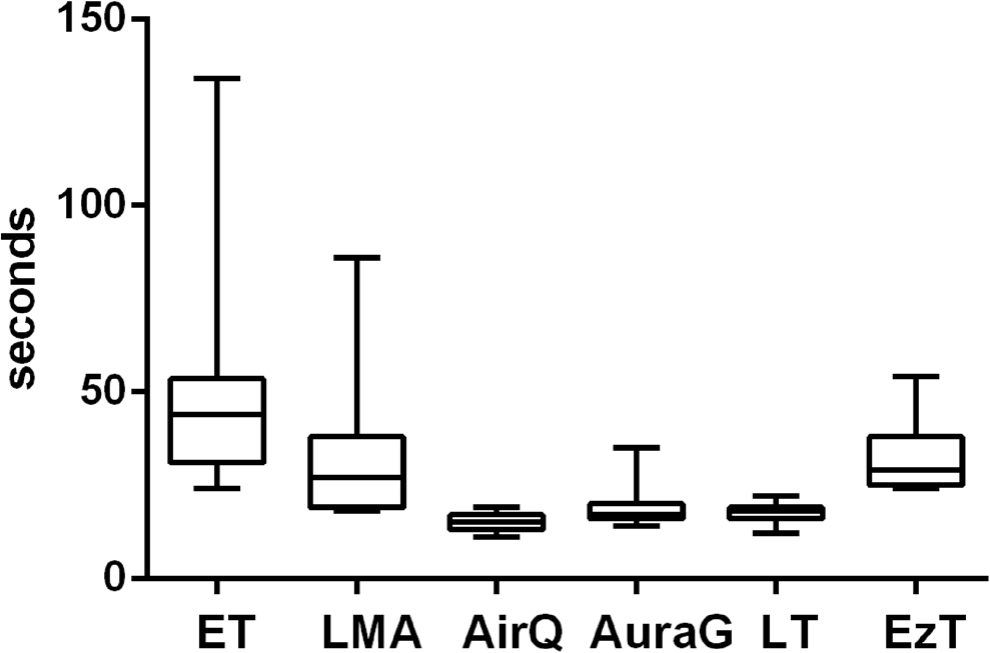
Time to final placement with tongue edema with ET, LMA, AirQ, AuraG, LT, and EzT

**Fig. 3:**
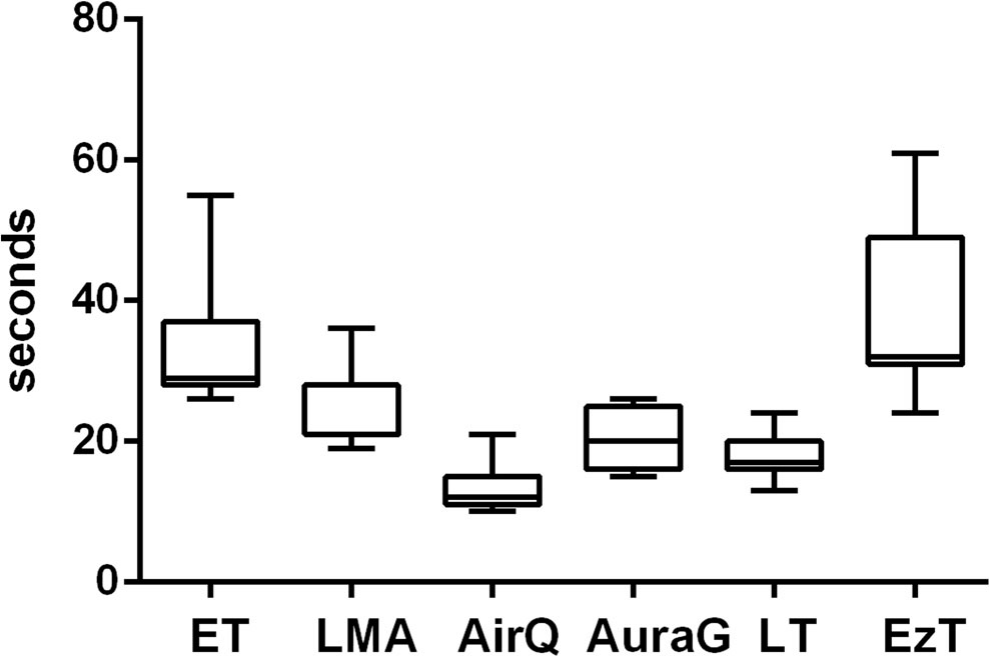
Time to final placement with limited mobility of the cervical spine with ET, LMA, AirQ, AuraG, LT, and EzT

The time to first ventilation varied under simulated physiologic conditions (Fig. 1). Under TE conditions, there were significantly longer insertion times for the ET, LMA, and EzT (Fig. 2). Under CS conditions, there were significantly longer insertion times for the ET, LMA, LT, and EzT (Fig. 3). A multivariate analysis showed experience with the respective device to be the only factor of influence on time to first ventilation.

### Skill retention

Under physiologic conditions, we found significant faster times to first ventilation with the ET, LT, and the EzT. Under TE conditions, all devices showed a significant training effect in terms of faster insertion times. Under CS conditions, we found significant improvements with the ET, AuraG, and the EzT.

### Improvement in success rate

The success rate under physiologic conditions increased significantly at the second run with the ET and the EzT (Table 2; rate of successful intubations < 30 s). During TE, again the ET and the EzT showed a significant increase in success rate. During CS, only the EzT showed significant improvement.

### Rating

**Fig. 4:**
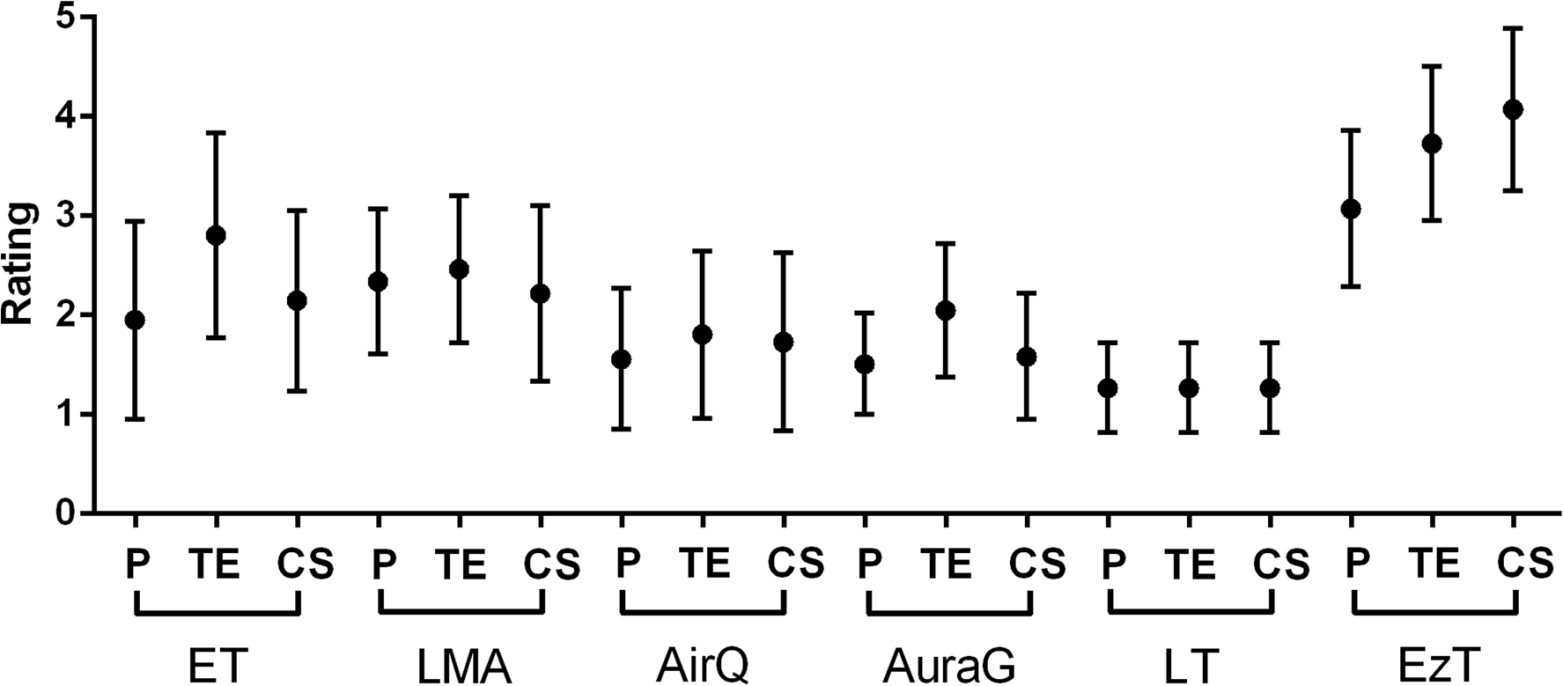
Rating under physiologic conditions (P), with tongue edema (TE), with limited mobility of the cervical spine (CS) with ET, LMA, AirQ, AuraG, LT, and EzT

Directly after the insertion attempts, participants were asked to rate the performance of the SADs under every given condition (1 = best, 5 = worst; Fig. 4, Table 3). Under simulated physiologic conditions, the participants rated the LT best, closely followed by the AuraG and the AirQ. Under simulated TE conditions, the LT was rated superiorly, followed by the AirQ. Under simulated CS condition, the LT was rated best, followed by the AuraG and the AirQ.

**Table 3:** Levels of significance under physiologic conditions (A) with tongue edema (B), and cervical immobilization (C) with endotracheal tube, laryngeal mask, AirQ, AuraGain, laryngeal tube, and EasyTube

	Endotracheal tube	Laryngeal mask	AirQ	AuraGain	Laryngeal tube	EasyTube
(A)						
Endotracheal tube		0.532	< 0.001	< 0.001	< 0.001	0.699
Laryngeal mask			< 0.001	0.009	0.007	0.485
AirQ				0.006	0.031	< 0.001
AuraGain					0.278	< 0.001
Laryngeal tube						< 0.001
EasyTube						
(B)						
Endotracheal tube		0.04	< 0.001	< 0.001	< 0.001	0.236
Laryngeal mask			< 0.001	< 0.001	< 0.001	0.274
AirQ				0.665	0.365	< 0.001
AuraGain					0.547	< 0.001
Laryngeal tube						< 0.001
EasyTube						
(C)						
Endotracheal tube		0.008	< 0.001	< 0.001	< 0.001	0.345
Laryngeal mask			< 0.001	0.452	0.01	< 0.001
AirQ				0.007	0.641	< 0.001
AuraGain					0.412	< 0.001
Laryngeal tube						< 0.001
EasyTube						

## Discussion

To our knowledge, this is the first study comparing the aforementioned SADs in simulated pediatric difficult airway situations. However, we have to keep in mind that there are only few SADs designed specifically for the pediatric airway. Also, one study compared other SADs in a pediatric manikin and some SADs were evaluated during pediatric anesthesia [5, 21].

The success rate under physiologic conditions with ET was comparable to other studies [4, 16]. With the SADs, success rates tend to differ largely, depending on the model and the setup [2, 19]. AirQ, AuraG, and LT were inserted the fastest in physiologic conditions and CS, most likely because these are easiest to handle. Under CS conditions, AirQ and LT showed superiority, with only 9.76% of the participants being able to insert the EzT within 30 s correctly. One disadvantage of the EzT in this context might be that its relatively stiff design might not be ideal for the small pediatric airway in this specific manikin. However, this exact feature has proven beneficial in another study [18]. Also, the EzT requires two cuffs to be inflated before ventilation.

The time difference under physiologic conditions differed between the devices; however, the time difference was clinically not relevant. Importantly, intubation with the ET and the EzT took the longest for the participants. Our data therefore support the statement of the ERC guidelines for resuscitation that only skilled and experienced practitioners should perform conventional ETI [14]. Also, more than two direct laryngoscopy attempts in children with difficult tracheal intubation are associated with a high failure rate and an increased incidence of severe complications [8]. The participants took more time to insert the EzT correctly, which might be owed to the fact that it was originally designed for adult patients.

As expected, all devices showed a significant training effect in terms of faster times, with a higher training effect in TE and CS. Also, success rates increased significantly for the ET and the EzT, as those two are presumable the most complicated to handle. If using these two devices in an emergency cart, frequent training is indispensable for proper performance, more than in the other tested devices. This is backed up by the observation that experience with the respective device was the only factor of influence on time to first ventilation.

Looking at ratings, LT, AuraG, and AirQ were rated best in all conditions, and all of them were close. Things that those devices have in common comprise ease of insertion, only one balloon to inflate, insertion without any additional device (e.g., laryngoscope), and simplicity in design. These three SADs also achieved the lowest times until first ventilation and the highest success rates. We therefore propose the use of one of these three devices in a pediatric emergency airway situation. We also surmised that novices preferred the easy technique and simplicity of supraglottic devices, unlike experts in intubation [17].

Strengths of our study comprise the variety of the tested devices, the comparison to ETI as the gold standard for securing an airway, the randomization of the SADs and the scenarios, and the simulation of the most common difficult airway settings (TE and CS). Also, the use of a manikin allows for reproducing the exact scenario for every participant. A manikin can be used to make similar practice sessions more realistic.

There are several limitations to our study. In particular, the use of only one type of manikin could have affected the insertion time and performance of each device [11]. Also, the participating colleagues had different experiences with each of the tested airways. The differences in professional education level and experience with ETI as well as SADs might also contribute to the varying results as shown. Furthermore, the time required for securing an airway in a manikin is generally shorter than in actual patients [9]. Simulated difficult airway situations in a manikin cannot simulate aggravated factors found in real-life pediatric emergencies, for example blood, swollen tonsils, or vomit which have influence on insertion time and success. The SimJunior pediatric simulator represents a 6-year-old. To date, there are no studies comparing this distinct manikin to human anatomy; a manikin’s anatomy may favor a specific model of airway device [11]. However, it has been shown before that the company’s adult manikin’s airway (SimMan) is acceptably realistic [10]. Also, manikins allow for simulating the exact same airway situations for each participant and pose the only way to simulate standardized airway situations to date.

## Conclusion

LT, AuraG, and AirQ were superior in providing fast and effective ventilation during simulated difficult airway situations in pediatricians with varying clinical experience.

## List of abbreviations

AuraG: AuraGain
CPR: Cardiopulmonary resuscitation
CS: Limited mobility of the cervical spine
ERC: European Resuscitation Council
ET: Endotracheal tube
EzT: EasyTube
ETI: Endotracheal intubation
LMA: Laryngeal mask airway
LT: Laryngeal tube
SAD: Supraglottic airway device
TE: Tongue edema
